# Potential biomarkers for retinopathy of prematurity identified by circular RNA profiling in peripheral blood mononuclear cells

**DOI:** 10.3389/fimmu.2022.953812

**Published:** 2022-08-23

**Authors:** Yun Li, Haixiang Zhou, Qian Huang, Wei Tan, Yuting Cai, Zicong Wang, Jingling Zou, Bingyan Li, Shigeo Yoshida, Yedi Zhou

**Affiliations:** ^1^ Department of Ophthalmology, The Second Xiangya Hospital of Central South University, Changsha, China; ^2^ Hunan Clinical Research Center of Ophthalmic Disease, Changsha, China; ^3^ Department of Ophthalmology, Kurume University School of Medicine, Kurume, Japan

**Keywords:** expression profile, microarray, diagnostic biomarker, retinopathy of prematurity, peripheral blood mononuclear cells, circular RNA

## Abstract

**Purpose:**

This study aims to reveal the altered expression profiles of circular RNAs (circRNAs) in the peripheral blood mononuclear cells (PBMCs) of patients with retinopathy of prematurity (ROP), and to identify potential biomarkers for ROP diagnosis.

**Methods:**

Differentially expressed circRNAs in PBMCs of five infants with ROP and five controls were identified using microarray analysis. Twelve altered circRNAs were validated using reverse transcription-quantitative real-time polymerase chain reaction (RT-qPCR). Bioinformatic analyses were conducted to predict the circRNA/miRNA interactions, competing endogenous RNA (ceRNA) network, related biological functions, and signaling pathways. Four selected circRNAs in PBMCs were verified using RT-qPCR in another cohort, including 24 infants with ROP and 23 premature controls, and receiver operating characteristic (ROC) curves were used to estimate their potential as diagnostic biomarkers of ROP.

**Results:**

A total of 54 and 143 circRNAs were significantly up- and down-regulated, respectively, in the PBMCs of patients with ROP compared with controls. Twelve of the significantly altered circRNAs were preliminarily validated by RT-qPCR, which confirmed the reliability of the microarray analysis. The circRNA/miRNA interactions and ceRNA network were displayed according to the altered circRNAs. Three circRNAs (hsa_circRNA_061346, hsa_circRNA_092369, and hsa_circRNA_103554) were identified as potential diagnostic biomarkers for ROP with certain clinical values.

**Conclusions:**

CircRNAs were significantly altered in PBMCs of treatment-requiring ROP patients. CircRNAs may be used as potential biomarkers and possible therapeutic targets for ROP.

## Introduction

Retinopathy of prematurity (ROP) is a major complication of premature birth and has become a significant problem with improved survival in premature infants (1). Severe cases of ROP may lead to long-term vision loss, and consistent with this ROP is a major cause of childhood blindness (2) so there is an urgent need to facilitate its diagnosis and treatment.

The diagnosis of ROP is usually based on fundus screening with binocular indirect ophthalmoscopy and/or a wide-field digital retinal imaging system (such as the RetCam digital retinal camera) (3). This screening method has low efficiency and a high rate of misdiagnosis, and associated treatment decisions are sometimes not objective. Thus, sensitive and specific biomarkers are needed for the diagnosis and assessment of prognosis in ROP (4).

While anti-vascular endothelial growth factor (VEGF) therapy and laser photocoagulation are effective and are used clinically for ROP treatment (5, 6), they have limitations (such as the requirement for repeated treatment) and are costly. Therefore, to identify novel therapeutic targets it is important to enhance understanding of the regulatory mechanisms of ROP pathogenesis.

As a novel class of endogenous non-coding RNAs, circular RNA (circRNA) has a closed structure without 5’ caps and 3’ poly-A ends (7), and is thus more stable than linear RNA (8). Therefore, circRNAs have great potential to be biomarkers in various diseases (9–11). Though incapable of coding proteins, circRNA functions as a molecular sponge by targeting microRNAs (miRNAs) and modulates mRNA expression by competing endogenous RNA (ceRNA) mechanisms (12, 13).

Previous studies have indicated that circRNAs have great potential for application in retinal disorders (14) including as diagnostic biomarkers for diabetic retinopathy (15, 16), retinoblastoma (17) and age-related macular degeneration (18). They are also considered to be therapeutic targets for retinal neovascular disease. For example, 26 differentially expressed circRNAs were identified in serum exosomes of patients with proliferative diabetic retinopathy, and a novel circRNA derived from high-glucose-induced endothelial cells, circFndc3b, has a regulatory role in angiogenesis (19). Yao et al. (20) also reported an anti-angiogenesis effect by targeting circRNA-MET *via* attenuation of endothelial tip cell specialization, while Deng et al. (21) demonstrated that CircPDE4B suppresses retinal pathological angiogenesis by sponging miR-181c and facilitating the ubiquitin degradation of HIF-1α. Despite these findings, the role played by circRNAs in regulating retinal neovascularization remains unclear.

We previously showed the altered expression profiles of circRNAs in a mouse model of oxygen-induced retinopathy (OIR) (22), which parallels the pathogenesis of ROP to some extent. However, circRNAs are tissue-, disease-, and species-specific (23), so further exploration is needed using clinical ROP samples.

An increasing number of studies have shown that ocular diseases are closely associated with the regulation of the systemic immune system. Silveira et al. revealed the relationship between up-regulated plasma levels of several cytokines (IL-6, IL-8, and TNF-α) at birth with the later development of treatment-requiring ROP (24). Gao et al. indicated that peripheral macrophage depletion significantly inhibits pathological retinal neovascularization in OIR model in mice (25). Activation of mononuclear phagocytes may participate in the pathogenesis and development of ischemic retinopathies including ROP, which provide a new promising target in preventing these diseases (26). As the key drivers of immune responses, peripheral blood mononuclear cells (PBMCs) are comprised of variety types of immune cells, such as lymphocytes (T cells, B cells and natural killer cells), monocytes and dendritic cells (27). PBMCs are widely used for transcriptomics analyses in clarifying the immunological mechanisms in different diseases (27). For example, circRNA expression profiles in PBMCs were revealed in patients with rheumatoid arthritis (9), multiple sclerosis (28) and systemic lupus erythematosus (29), which identified potential biomarkers and predicted possible immunological mechanisms of these diseases.

The aim of this study was to identify novel potential biomarkers and possible molecular targets. We conducted microarray analysis with verification by reverse transcription quantitative real-time polymerase chain reaction (RT-qPCR) in the PBMCs of patients with ROP. The potential values of the altered circRNAs were assessed in an expanded cohort, and further bioinformatic analyses were performed to identify those possibly involved in biological functions and signaling pathways.

## Materials and methods

### Study subjects

In total, 57 preterm newborns were included in this study between December 2020 and January 2022 at the Second Xiangya Hospital of Central South University, Changsha, China. Among them, 29 infants had a diagnosis of ROP and required therapeutic treatment (five for screening and 24 for validation) according to the International Classification of Retinopathy of Prematurity protocol (30). The remaining 28 preterm newborns without retinopathy were enrolled as controls (five for screening and 23 for validation). The study subjects were recruited with exclusion criteria as reported (31). Blood was collected prior to treatment. For ethical reasons, to avoid additional blood draw, samples were collected from the control group at their last blood draw before leaving the neonatal intensive care unit. The study protocol was approved by the Ethics Committee of the Second Xiangya Hospital of Central South University and adhered to the tenets of the Declaration of Helsinki. Informed consent was obtained from the participants’ guardians. The clinical characteristics of the included subjects are summarized in **Table 1**.

**Table 1:** Clinical characteristics of the individuals included in this study.

Characteristics	Screening cohort			Validation cohort		
	Control (n=5)	ROP (n=5)	P-value	Control (n=23)	ROP (n=24)	P-value
Gestational age, mean ± SD, weeks	30.94 ± 1.09	28.29 ± 2.08	0.035	31.24 ± 1.28	28.54 ± 2.43	<0.001
Birth weight, mean ± SD, g	1314.00 ± 121.16	1080.00 ± 258.84	0.105	1454.78 ± 263.58	1137.50 ± 379.65	0.002
Postmenstrual age at blood draw, mean ± SD, weeks	37.26 ± 0.16	37.83 ± 0.89	0.195	36.68 ± 1.52	39.99 ± 3.68	<0.001
Body weight at blood draw, mean ± SD, g	2236.00 ± 119.29	2252.00 ± 113.67	0.834	2247.61 ± 266.03	2810.83 ± 960.63	0.012
Sex (male/female)	1/4	3/2	0.197	9/14	12/12	0.454

SD, standard deviation; ROP, retinopathy of prematurity.

### Preparation of PBMCs and RNA isolation

1.0–1.5 ml of venous blood from each study subject was collected in an anticoagulant tube (Ethylene Diamine Tetraacetic Acid) in the morning and was taken to the laboratory within 2 h. PBMCs were isolated from the blood by density gradient centrifugation using Ficoll-Paque PLUS (GE Healthcare, NJ, USA). Total RNAs were extracted using TRIzol reagent (Invitrogen, Carlsbad, USA), and the samples were stored at -80°C. The NanoDrop ND-1000 (Thermo Scientific, Wilmington, DE, USA) was used to measure the concentrations. The RNA integrity was assessed by electrophoresis on a denaturing agarose gel.

### Microarray analysis of circRNAs

Five infants with ROP needing treatment and five controls were included for the microarray analysis which was conducted as previously described (9). In brief, prepared total RNAs were treated with Rnase R (Epicentre, Madison, WI, USA) to enrich circRNAs, and were then amplified and transcribed into fluorescent cRNA using random primer and an Arraystar Super RNA Labeling Kit (Arraystar, Rockville, MD, USA). The labeled cRNAs were hybridized onto the Arraystar Human circRNA Arrays V2 (8x15K, Arraystar), and incubated in an Agilent Hybridization Oven. After washing, the slide scanning was conducted by using the Agilent Scanner G2505C. The images were analyzed using Agilent Feature Extraction software (version 11.0.1.1). The criterion for significant alteration of circRNAs was ≥2-fold increase or decrease and *P*<0.05. The circRNA microarray analysis raw data were deposited in the Gene Expression Omnibus database (Accession No. GSE204780).

### RT-qPCR

To validate the results of microarray analysis, RT-qPCR was performed as previously described (32) with slight modification. In brief, total RNAs were transcribed into cDNAs using the SuperScript III Reverse Transcriptase kit (Invitrogen, Carlsbad, CA, USA). RT-qPCR was performed using a QuantStudio5 Real-time PCR System (Applied Biosystems, Foster City, CA, USA) with 2× PCR Master Mix (Arraystar). The sequences of the primers are listed in **Table 2**. The relative expression level of circRNAs were normalized to those of β-actin.

**Table 2:** List of primer sequences of the circRNAs for real-time polymerase chain reaction (RT-qPCR).

Name	Primer sequence	Tm (°C)	Product length (bp)
β-actin(human)	F:5’ GTGGCCGAGGACTTTGATTG3’ R:5’ CCTGTAACAACGCATCTCATATT3’	60	73
hsa_circRNA_003986	F:5’ CCGAGTTTGTAATGTGACTAGACG3’ R:5’ ATTGGCTAACAGTCGAATTGGT3’	60	122
hsa_circRNA_007366	F:5’ GCTGGAGGTCGATATTGATTAC3’ R:5’ ATTCTGAAACTCATTCCCCTTG3’	60	58
hsa_circRNA_020959	F:5’ TTCTGCTGAGAGATCACCTCC 3’ R:5’ AATGGATTGCCAAAACGGTC3’	60	57
hsa_circRNA_061346	F:5’ GAAGTGTGCCCCATTCTTTTAC3’ R:5’ TTCGCAAACATCCATCCTCT3’	60	172
hsa_circRNA_082319	F:5’ TCTTGGCGCACAAACAGTCTA 3’ R:5’ CAACATCCCAAATCGGTCTG3’	60	148
hsa_circRNA_092369	F:5’ GTATGCATACTACCTTGTACTGGTT3’ R:5’ GACTATTGAAACCTGGAGAAACT3’	60	154
hsa_circRNA_103399	F:5’ CTCCTCTCAAACCCAAACTCAA3’ R:5’ CTTCAGGTTCCCATCCACAAT3’	60	83
hsa_circRNA_103555	F:5’ GACAATGCTGCTTTCCCTTTC 3’ R:5’ CCAGTAACCGGATGCTTCACA 3’	60	154
hsa_circRNA_103556	F:5’ AACTGAGTGTGAGAGACTGGCA 3’ R:5’ GGTTCTCCACCAAATTGATG3’	60	164
hsa_circRNA_103557	F:5’ ATGGGACTATTGCTGTGATCGT3’ R:5’ ACTGCCACACAGAAGAACCAAT 3’	60	54
hsa_circRNA_003140	F:5’ GACAGTTGTTACCAGTGAGCCTT3’ R:5’ GCACTCGTGCATAACTATTACTGA 3’	60	123
hsa_circRNA_103554	F:5’ CTGAACCAATACAGAGCAGACAT3’ R:5’ GAACTGCCACACAGAAGAACTC3’	60	188

### Bioinformatics analysis

Predictions of circRNA-miRNA interactions were conducted using Arraystar’s miRNA target prediction software according to TargetScan and miRanda. The circRNA-miRNA-mRNA network was constructed according to the competing endogenous RNA (ceRNA) hypothesis. Gene Ontology (GO) and Kyoto Encyclopedia of Genes and Genomes (KEGG) pathway analyses were used for further predictions of involvement in biological functions and signaling pathways.

### Statistical analysis

Numeric variables were compared using a *Student’s t*-test or *Mann-Whitney U*-test, and categorical variables were compared using a Chi-square test. *P*<0.05 was considered statistically significant. Moreover, to adjust for multiple comparisons, FDR (false discovery rate) is calculated with Benjamini-Hochberg method.

## Results

### Expression profiling of circRNAs in PBMCs from patients with ROP

**Figure 1:**
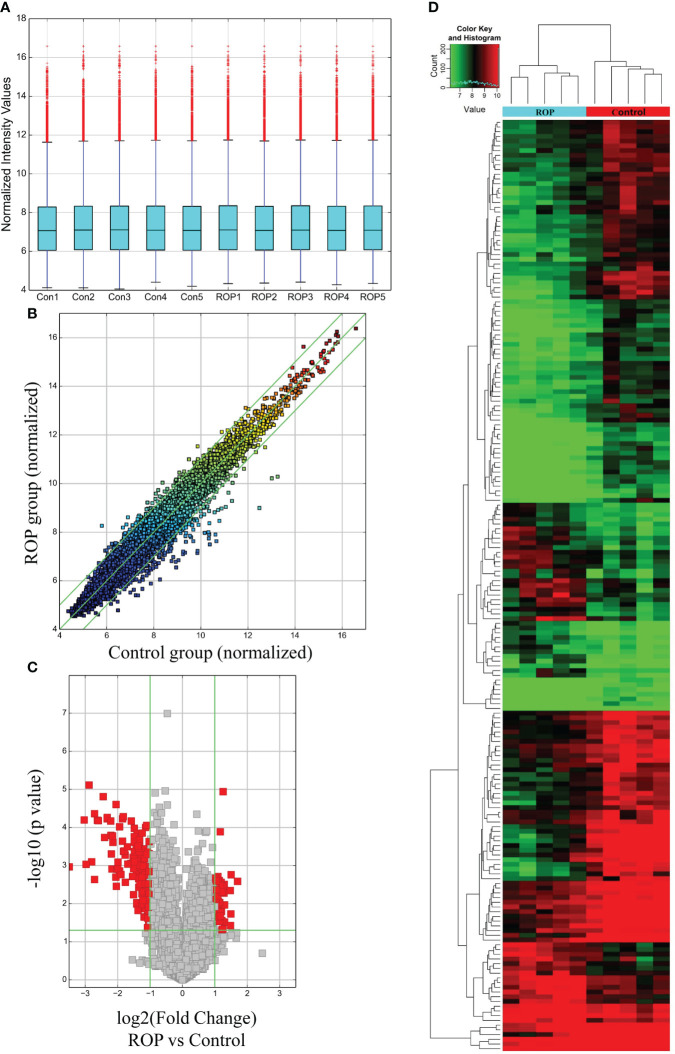
Expression profile of circRNAs in retinopathy of prematurity (ROP) patients and controls. **(A)** A box plot shows the expression profile of circRNAs in each blood sample after normalization. **(B)** Raw variations of the circRNA expression profile are displayed on a scatter plot. **(C)** A volcano plot shows significantly altered circRNAs using a threshold of ≥2-fold change and P<0.05. **(D)** Heatmap derived from hierarchical cluster analysis showing different expressions of circRNAs in each sample.

As demonstrated by the box plot (**Figure 1A**), all samples showed stable and similar distributions of circRNA profiles. Variations in the expressed circRNAs are indicated by a scatter plot (**Figure 1B**) and a volcano plot (**Figure 1C**), and differentially expressed circRNAs were identified and classified as shown by the heatmap (**Figure 1D**). Using a threshold value of ≥2-fold change and *P*<0.05, a total of 54 circRNAs were significantly up-regulated and 143 circRNAs were down-regulated in the PBMC samples of patients with ROP compared to the controls. Among them, two up-regulated and 19 down-regulated circRNAs met FDR<0.05.

The top 10 up- and down-regulated circRNAs are summarized in **Table 3**, with hsa_circRNA_088200 and hsa_circRNA_020959 showing the greatest increase or decrease, respectively.

**Table 3:** Top 10 up- and down-regulated circRNAs identified by microarray analysis in peripheral blood mononuclear cells (PBMCs) of infants with retinopathy of prematurity (ROP) compared with premature controls.

circRNA	chrom	circRNA_type	GeneSymbol	FC (abs)	Regulation	P-value	FDR
hsa_circRNA_088200	chr9	exonic	TNC	3.256831	up	0.002593	0.136782
hsa_circRNA_091000	chrX	exonic	NONO	2.835938	up	0.001744	0.124536
hsa_circRNA_001715	chr7	exonic	LIMK1	2.820852	up	0.018692	0.255727
hsa_circRNA_105034	chrX	exonic	AFF2	2.800486	up	0.037582	0.310382
hsa_circRNA_092368	chr1	sense overlapping	CNN3	2.783897	up	0.004532	0.164641
hsa_circRNA_071935	chr5	exonic	TRIO	2.467981	up	0.010800	0.215514
hsa_circRNA_104016	chr5	exonic	ERGIC1	2.467952	up	0.030440	0.293641
hsa_circRNA_403982	chr8	exonic	ARHGEF10	2.452006	up	0.003123	0.143279
hsa_circRNA_003140	chr15	exonic	SPRED1	2.435171	up	0.005285	0.173989
hsa_circRNA_405520	chr17	exonic	ABR	2.391163	up	0.000012	0.033449
hsa_circRNA_020959	chr11	exonic	HBG1	11.256796	down	0.001087	0.101860
hsa_circRNA_092535	chr3	intronic	TFRC	8.181255	down	0.000064	0.037264
hsa_circRNA_020964	chr11	exonic	HBG2	7.854892	down	0.000932	0.093134
hsa_circRNA_103555	chr3	exonic	TFRC	7.368595	down	0.000008	0.033449
hsa_circRNA_020960	chr11	exonic	HBG1	6.964511	down	0.000800	0.089225
hsa_circRNA_068601	chr3	exonic	TFRC	6.552692	down	0.000044	0.037264
hsa_circRNA_100436	chr1	exonic	TMCC2	6.547898	down	0.002326	0.134515
hsa_circRNA_092369	chr1	exonic	TMEM56	6.166317	down	0.000065	0.037264
hsa_circRNA_037139	chr16	exonic	HBA2	6.042306	down	0.000066	0.037264
hsa_circRNA_103556	chr3	exonic	TFRC	5.446337	down	0.000016	0.035010

FDR, false discovery rate (Benjamini-Hochberg method).

### GO enrichment and KEGG pathway analyses of the significantly altered circRNAs

**Figure 2:**
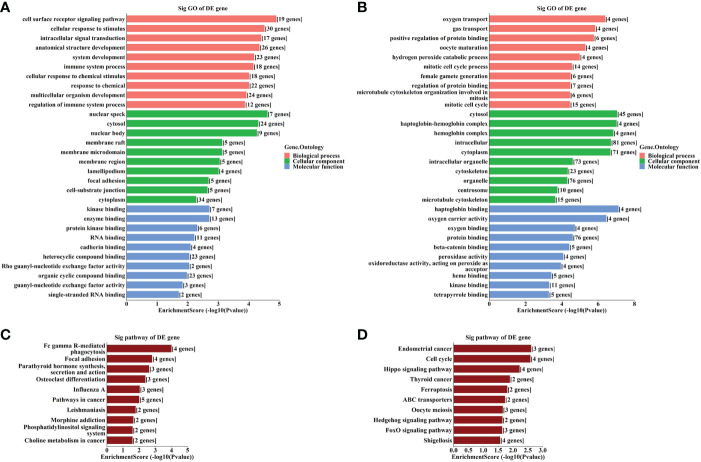
GO and KEGG analyses according to the host genes of the significantly altered circRNAs. **(A)** Top gene ontology (GO) terms with the host genes of the up-regulated circRNAs. **(B)** Top GO terms with the host genes of the down-regulated circRNAs. **(C)** Top Kyoto Encyclopedia of Genes and Genomes (KEGG) pathways with the host genes of the up-regulated circRNAs. **(D)** Top KEGG pathways with the host genes of the down-regulated circRNAs.

The results of GO analysis showed that the host genes of the up-regulated circRNAs were most enriched in the “cell surface receptor signaling pathway”, “nuclear speck” and “kinase binding” (**Figure 2A**), and those of the down-regulated circRNAs were most enriched in “oxygen transport”, “cytosol” and “haptoglobin binding” (**Figure 2B**). Meanwhile, KEGG pathway analysis indicated that the up- and down-regulated circRNAs may be involved in “Fc gamma R-mediated phagocytosis” and “cell cycle”, respectively (**Figures 2C, D**).

### Preliminary verification of the altered circRNAs

**Figure 3:**
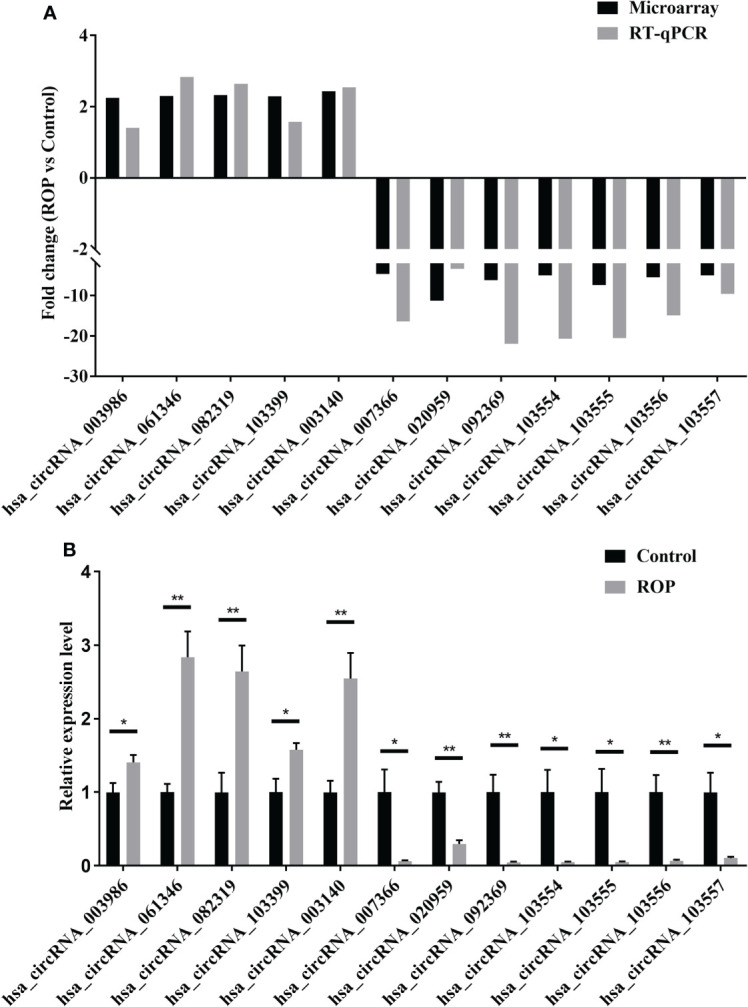
Preliminary verification of twelve differentially expressed circRNAs. **(A)** Fold changes of these circRNAs by microarray and real-time polymerase chain reaction (RT-qPCR). **(B)** Relative expression levels of these circRNAs assessed by RT-qPCR. n=5 for each group. ROP, retinopathy of prematurity. *, P<0.05, **, P<0.01.

Results of RT-qPCR (**Figure 3A**) showed that five circRNAs (hsa_circRNA_003986, hsa_circRNA_061346, hsa_circRNA_082319, hsa_circRNA_103399, and hsa_circRNA_003140) were significantly up-regulated and seven (hsa_circRNA_007366, hsa_circRNA_020959, hsa_circRNA_092369, hsa_circRNA_103554, hsa_circRNA_103555, hsa_circRNA_103556, and hsa_circRNA_103557) were significantly down-regulated (**Figure 3B**).

### Prediction of circRNA-miRNA interactions

**Figure 4:**
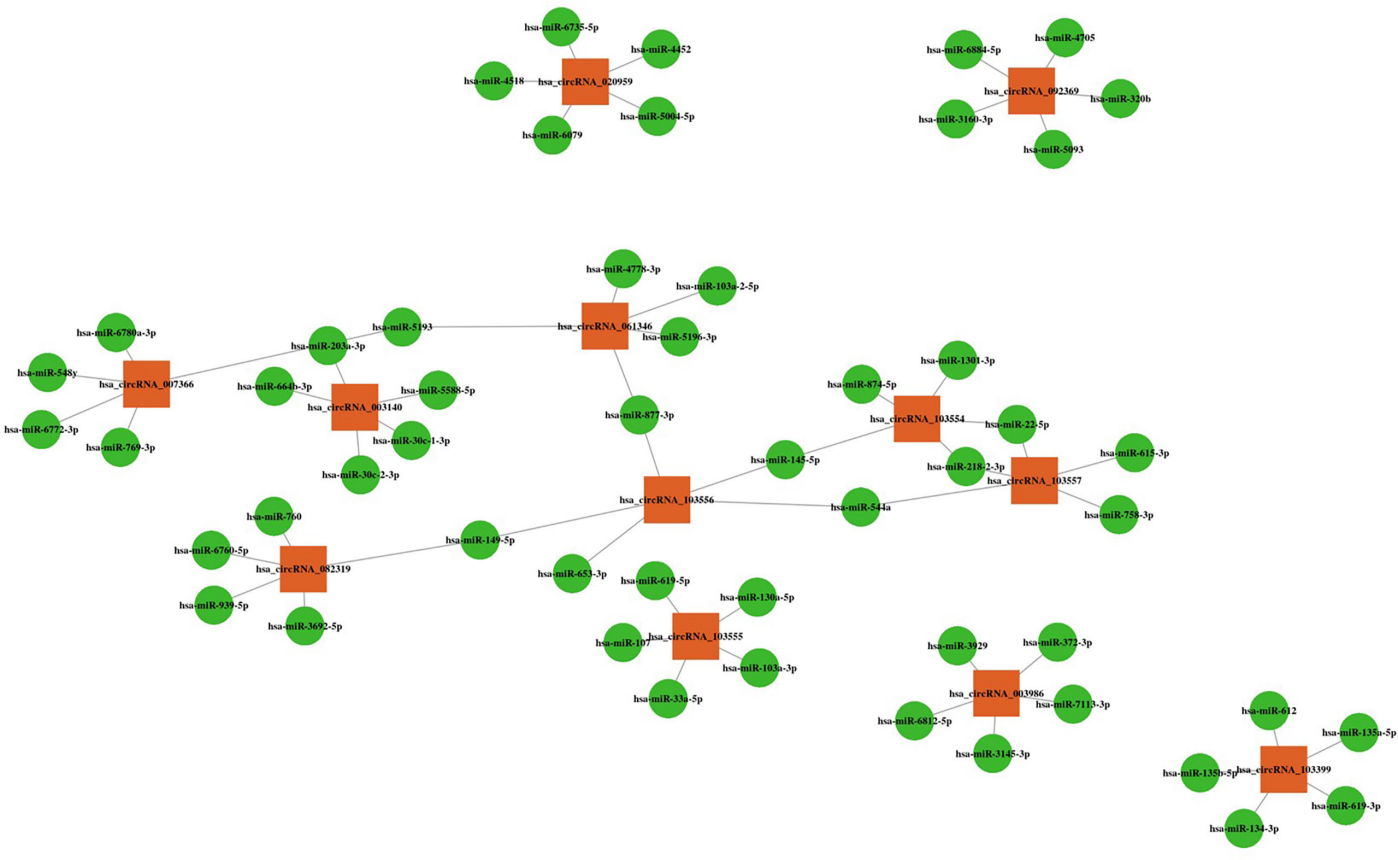
CircRNA-miRNA interactions with the top five targeted miRNAs. Orange squares represent altered circRNAs, and green circles represent target miRNAs.

Bioinformatics analysis revealed five miRNAs associated with each altered circRNA. The top five miRNAs are shown in **Figure 4**.

### Competing endogenous RNA (ceRNA) regulatory network

**Figure 5:**
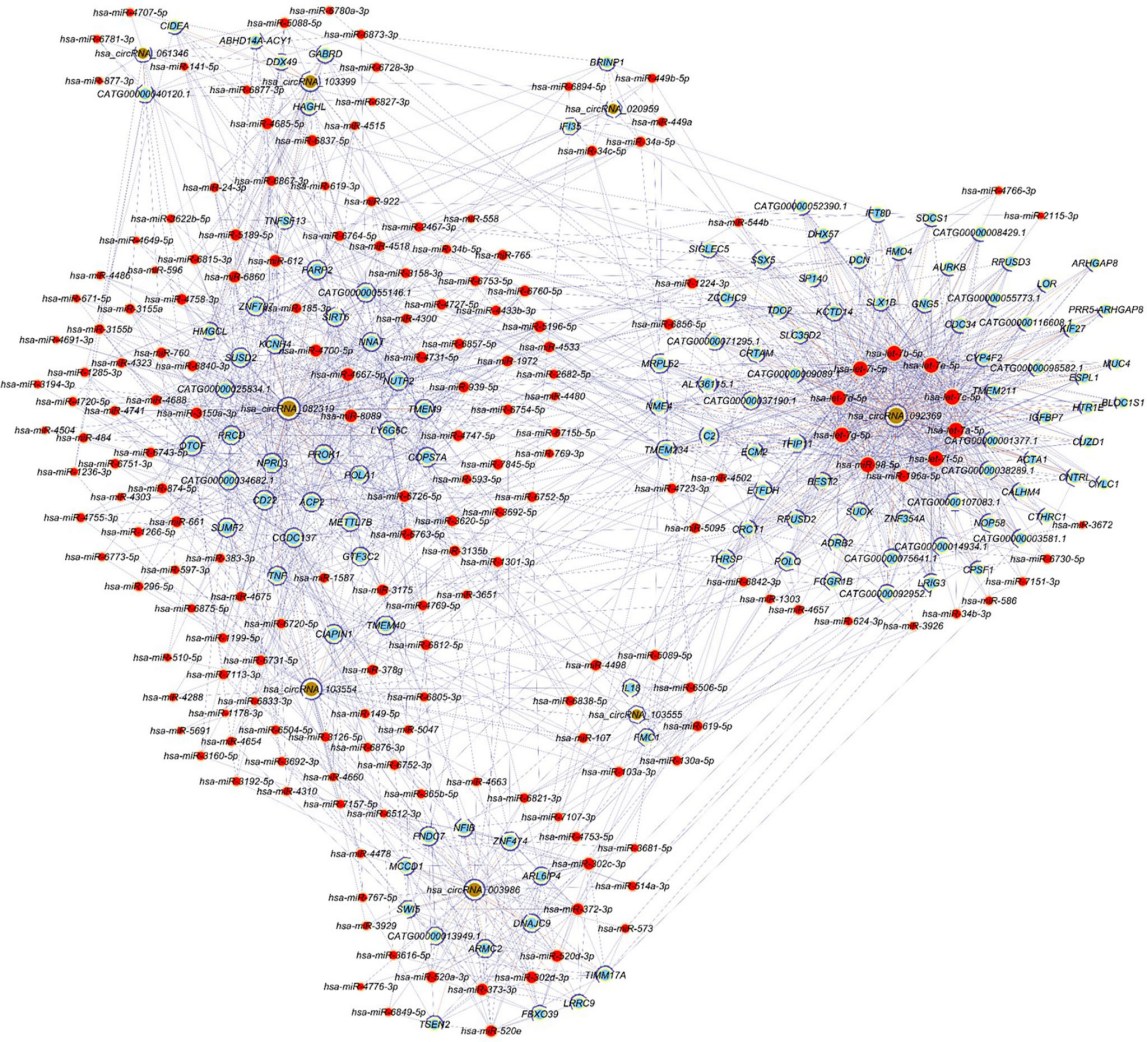
CircRNA-miRNA-mRNA regulatory network. Red nodes represent miRNAs, light-blue nodes represent target genes, and brown nodes represent circRNAs. Edges with T-shape arrow and edges without arrow represent directed and undirect relationships, respectively.

**Figure 5** shows the circRNA-miRNA-mRNA network constructed according to ceRNA regulation. The network includes 314 nodes (eight circRNAs, 184 miRNAs and 122 target genes) and 1862 edges.

### GO enrichment and KEGG pathway analyses of the target genes

**Figure 6:**
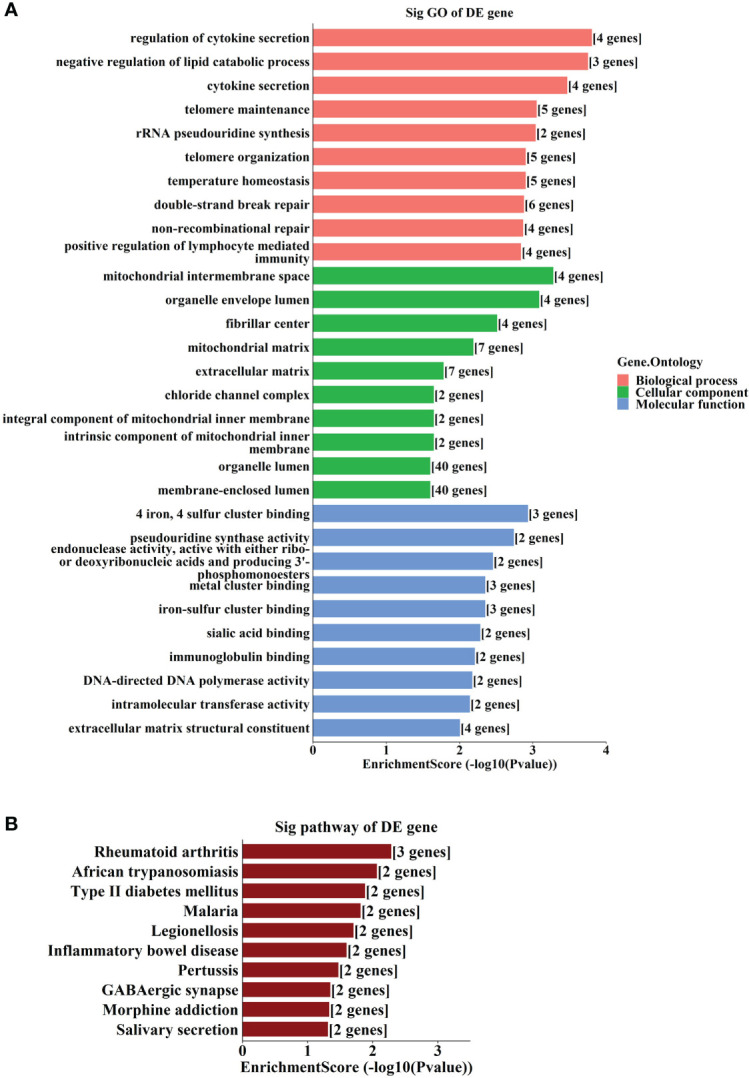
GO and KEGG analyses according to the target genes in ceRNA network. **(A)** Top gene ontology (GO) terms with the target genes in the competing endogenous RNA (ceRNA) network. **(B)** Top Kyoto Encyclopedia of Genes and Genomes (KEGG) pathways with the target genes in ceRNA network.

GO and KEGG analyses showed that the most enriched GO terms include “regulation of cytokine secretion”, “mitochondrial intermembrane space”, and “4 iron, 4 sulfur cluster binding” (**Figure 6A**). The most enriched KEGG pathways include “Type II diabetes mellitus” and “GABAergic synapse” (**Figure 6B**).

### Validation of potential biomarkers and their clinical values

**Figure 7:**
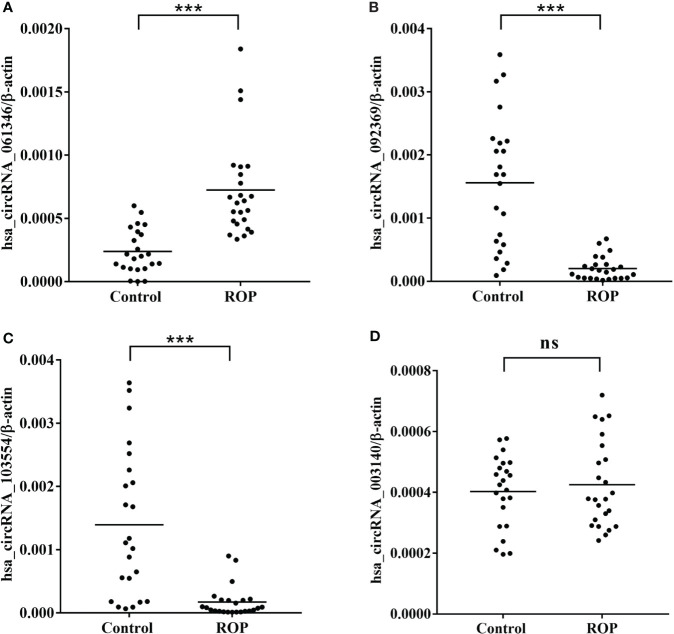
Real-time polymerase chain reaction (RT-qPCR) validation of hsa_circRNA_061346 **(A)**, hsa_circRNA_092369 **(B)**, hsa_circRNA_103554 **(C)**, and hsa_circRNA_003140 **(D)** in a larger cohort with 24 infants with retinopathy of prematurity (ROP) and 23 controls. ***, P< 0.001. ns, not significant.

The results of RT-qPCR analysis of four circRNAs (hsa_circRNA_061346, hsa_circRNA_092369, hsa_circRNA_103554 and hsa_circRNA_003140) in a larger cohort with (24 ROP infants and 23 controls) showed significantly increased expression of hsa_circRNA_061346 and significantly decreased expressions of hsa_circRNA_092369 and hsa_circRNA_103554 in the ROP group (**Figures 7A–C**, P<0.001). However, no significant change was found in expression of hsa_circRNA_003140 (**Figure 7D**, P>0.05).

**Figure 8:**
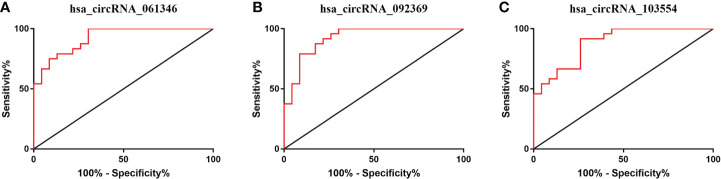
Receiver operating characteristic (ROC) curve analyses of hsa_circRNA_061346 **(A)**, hsa_circRNA_092369 **(B)**, and hsa_circRNA_103554 **(C)** in patients with retinopathy of prematurity.

To assess the sensitivity and specificity of these three statistically altered circRNAs, we performed ROC curve analysis, and the areas under the curve (AUC) was calculated (**Figure 8**). The AUC values for hsa_circRNA_061346, hsa_circRNA_092369 and hsa_circRNA_103554 were 0.9239, 0.9239 and 0.8822 respectively (**Table 4**). Moreover, we determined the cutoff values of hsa_circRNA_061346, hsa_circRNA_092369 and hsa_circRNA_103554 as >0.00047, <0.0002775 and <0.0005235, indicating that diagnostic sensitivities/specificities were 75.00/91.30, 79.17/91.30, and 91.67/73.91 (%), respectively (**Table 4**).

**Table 4:** Diagnostic values of the potential biomarkers of the patients with retinopathy of prematurity (ROP). .

Index (circRNA)	Cutoff value (circRNA/β-actin)	Sensitivity (%)	Specificity (%)	AUC	95% confidence interval of AUC	P-value
hsa_circRNA_061346	>0.00047	75.00	91.30	0.9239	0.8532 to 0.9946	<0.0001
hsa_circRNA_092369	<0.0002775	79.17	91.30	0.9239	0.8485 to 0.9993	<0.0001
hsa_circRNA_103554	<0.0005235	91.67	73.91	0.8822	0.7893 to 0.9752	<0.0001

## Discussion

The pathogenesis of ROP, a major cause of childhood blindness, remains unclear. As a novel subtype of ncRNAs, circRNAs serve as promising biomarkers as well as potential targets in a variety of ophthalmic diseases (33). In this study, we identified circRNAs with altered expression levels in ROP. As a type of non-coding RNAs, circRNAs have no protein-coding capability, alternatively, we may reveal the possible mechanisms of from the host genes of those identified circRNAs. For example, hsa_circRNA_103554 has been validated to be lower expressed in the ROP group (**Figure 7**), and transferrin receptor (TFRC) is the host gene. Transferrin receptor 1 (TfR1), encoded by TFRC, is a key modulator of iron homeostasis and regulate the pathogenesis in a variety of disorders (34). Deletion of TfR1 inhibits angiogenesis through reduction of mitochondrial complex I in limb ischemia in mice (35). The roles played by hsa_circRNA_103554 and its host gene TFRC in the pathogenesis of hypoxia-induced retinal neovascularization deserve to be further studied.

Using GO analysis, we found that the host genes of the down-regulated circRNAs were enriched in oxygen transport and binding. ROP is induced by hypoxia leading to pathological neovascularization, and oxygen plays an important role in the pathogenesis of ROP (36). An *in vivo* study indicated that hyperoxia treatment is a useful therapeutic strategy in targeting pathological neovascularization in ischemic retinopathy, and does not have the inflammatory effect related to anti-VEGF therapies (37). A novel oxygen management strategy has been found to decrease the rate of ROP (38). Future studies may be needed to further investigate the roles and mechanisms of circRNAs in oxygen transport in ROP.

It has been shown that circRNA acts as a molecular sponge by binding to miRNAs (39), and in the present study we found interactions between miRNA and the altered circRNAs. Some miRNAs interacted with more than one circRNA, for example, hsa_circRNA_082319 and hsa_circRNA_103556 shared the same miRNA (hsa-miR-149-5p), indicating that these molecules may play a joint regulatory role in the pathological process. Competing endogenous RNA is constituted by coding-protein mRNAs, miRNAs and circRNAs, and regulates gene expressions by competitively binding to common miRNAs (40). The ceRNA regulatory network constructed in the current study identified numerous miRNAs and target genes, and GO and KEGG analyses identified key biological processes such as “regulation of cytokine secretion”. Many cytokines, such as IL-12, IL-17 and IL-23 have been found to have fundamental roles in ocular angiogenesis and ROP pathogenesis (36, 41–43). Therefore, the present results of bioinformatics analysis are consistent with previous findings, and indicate that circRNAs may play a regulatory role in the effect of cytokines on angiogenesis.

Recent studies have also revealed several novel functions of circRNAs in physiological and pathological conditions, such as translation (44, 45), binding proteins (46), and m^6^A methylation (47), which deserve to be further studied.

We found that hsa_circRNA_061346, hsa_circRNA_092369 and hsa_circRNA_103554 are promising biomarkers in diagnosis of treatment-requiring ROP (AUC > 0.88)

This study provides new findings on the alteration of circRNAs in PBMCs of ROP patients, and suggests several potential biomarkers for ROP diagnosis. However, the study is limited by its small sample size and the lack of ROP patients not requiring treatment. Moreover, there are statistically significant differences in the clinical characteristics, such as gestational age (**Table 1**), which might affect the results of the assessment. Future clinical studies with larger subjects are needed to verify the clinical values of these potential biomarkers. Besides, in the current study, β-actin has been used as the reference gene for RT-qPCR. Although it has been widely used for normalization in RT-qPCR, numerous studies suggested using multiple reference genes to achieve more convincing results with better stability (48–50). And this should also be considered in future validation studies.

In conclusion, circRNAs were significantly altered in PBMCs of treatment-requiring ROP patients. Three circRNAs were identified and validated to be promising potential biomarkers ROP diagnosis. The significantly expressed circRNAs identified in this study might also be considered as possible therapeutic targets in the treatment of ROP, while further investigations are necessary to explore the exact roles and mechanisms of those specific circRNAs.

## Data availability statement

The datasets presented in this study can be found in online repositories. The names of the repository/repositories and accession number(s) can be found below: Gene Expression Omnibus database (Accession No. GSE204780).

## Ethics statement

The studies involving human participants were reviewed and approved by the ethics committee of the Second Xiangya Hospital of Central South University. Written informed consent to participate in this study was provided by the participants’ legal guardian/next of kin.

## Author contributions

YZ conceived and designed the study. YL, HZ, QH, WT, YC, and ZW obtained the clinical records, collected, and prepared the samples, YZ, JZ, BL, and SY conducted the data analyses. YL and YZ wrote the manuscript. All authors contributed to the article and approved the final version of the manuscript.

## Funding

This work was supported by Changsha Science and Technology Project (kq1907075), National Natural Science Foundation of China (No. 81800855, 82171087), Scientific Research Project of Hunan Provincial Health Commission (No. 202207022574) and New Technology Incubation Funds in Ophthalmology.

## Conflict of interest

The authors declare that the research was conducted in the absence of any commercial or financial relationships that could be construed as a potential conflict of interest.

## Publisher’s note

All claims expressed in this article are solely those of the authors and do not necessarily represent those of their affiliated organizations, or those of the publisher, the editors and the reviewers. Any product that may be evaluated in this article, or claim that may be made by its manufacturer, is not guaranteed or endorsed by the publisher.
